# RSCanner: rapid assessment and visualization of RNA structure content

**DOI:** 10.1093/bioinformatics/btad111

**Published:** 2023-03-01

**Authors:** Gandhar Mahadeshwar, Rafael de Cesaris Araujo Tavares, Han Wan, Zion R Perry, Anna Marie Pyle

**Affiliations:** Department of Molecular Biophysics and Biochemistry, Yale University, New Haven, CT 06511, United States; Department of Chemistry, Yale University, New Haven, CT 06511, United States; Department of Molecular, Cellular and Developmental Biology, Yale University, New Haven, CT 06511, United States; Department of Molecular Biophysics and Biochemistry, Yale University, New Haven, CT 06511, United States; Department of Chemistry, Yale University, New Haven, CT 06511, United States; Department of Molecular, Cellular and Developmental Biology, Yale University, New Haven, CT 06511, United States; Howard Hughes Medical Institute, Chevy Chase, MD 20815, United States

## Abstract

**Motivation:**

The increasing availability of RNA structural information that spans many kilobases of transcript sequence imposes a need for tools that can rapidly screen, identify, and prioritize structural modules of interest.

**Results:**

We describe *R*NA *S*tructural *C*ontent Sc*anner* (RSCanner), an automated tool that scans RNA transcripts for regions that contain high levels of secondary structure and then classifies each region for its relative propensity to adopt stable or dynamic structures. RSCanner then generates an intuitive heatmap enabling users to rapidly pinpoint regions likely to contain a high or low density of discrete RNA structures, thereby informing downstream functional or structural investigation.

**Availability and implementation:**

RSCanner is freely available as both R script and R Markdown files, along with full documentation and test data (https://github.com/pylelab/RSCanner).

## 1 Introduction

The advent of powerful platforms for RNA structure interrogation (Smola et al. 2015) has generated large amounts of structural data for individual RNAs and even whole transcriptomes (Zubradt et al. 2017). We can now visualize RNA secondary structure models, which can be used as roadmaps for planning downstream functional studies on individual transcripts. Given our enhanced ability to accurately model the structures of RNAs that are several kilobases in length (Huston et al. 2021), there is an increased need to prioritize structural features within new RNA targets and to identify stable regions of fixed base-pair content (BPC). RNA regions that sample a limited number of stable structural conformers are good candidates for structure–function studies and subsequent 3D elucidation (Akiyama et al. 2021). In contrast, unstructured and conformationally variable regions can also play important functional roles, and they may serve as useful probe hybridization sites (McHugh and Guttman 2018). Despite the availability of tools for detecting structured RNA regions derived from experimental data (Quarrier et al. 2010; Smola et al. 2015; Gilmore et al. 2017; Incarnato et al. 2018), there is currently no program that is able to quantify and rank the relative levels of RNA secondary structure *de novo*based on base-pairing probabilities.

We previously introduced a strategy for computing stable BPC along the length of long RNA molecules such as viral genomes, thereby providing a means for selecting transcript modules for subsequent structural investigation (Tavares et al. 2021). In the current work, we present *R*NA *S*tructural *C*ontent Sc*anner* (RSCanner), an R package that automates and extends the previous methodology by including customized parameters and standardized outputs. RSCanner identifies and analyzes modules of high BPC across any RNA target of interest, regardless of length. RSCanner scans an RNA secondary structure model in windows, calculates relative BPC, and applies a Shannon entropy (SE) filter derived from base-pairing probabilities to identify regions where a high degree of secondary structure coincides with regions that are conformationally restricted or conformationally variable. In doing so, RSCanner enables the user to quickly pinpoint regions of high and low density of discrete RNA structures, which can significantly facilitate the choice of candidate regions for biochemical and structural analysis, as well as aid in downstream experimental design.

## 2 Overview of RSCanner

**Figure 1:**
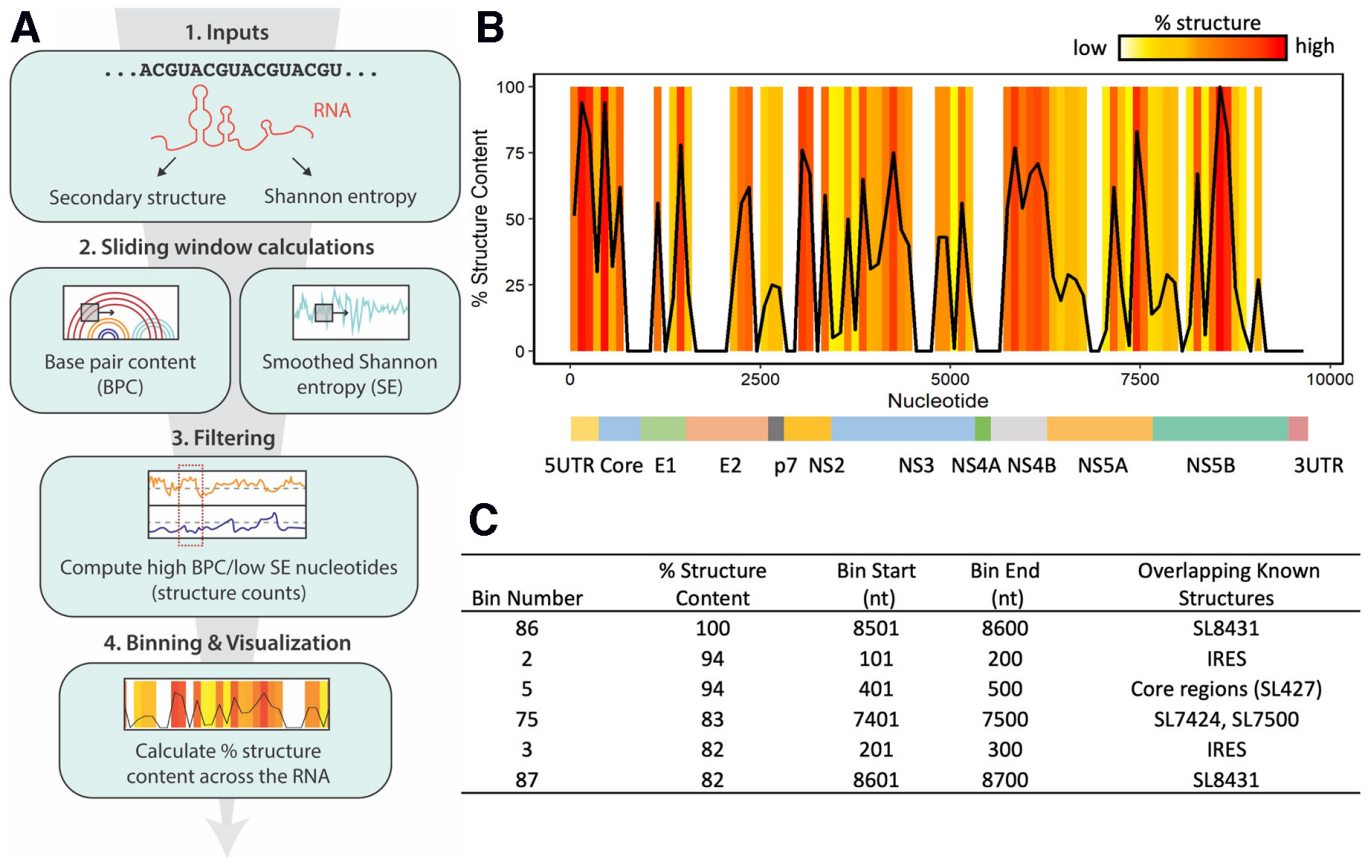
RSCanner workflow and application to the HCV RNA genome. (A) Flowchart of RSCanner algorithmic steps. (B) Final graphic output of RSCanner analysis on the HCV RNA genome. Note that the HCV genomic secondary structure that was used as input (Step 1, A) was obtained experimentally, as described in Wan et al. (2022). The boundaries of HCV coding regions are shown under the plot. The plot is colored based on the percentage of structure content. (C) Table showing RSCanner data output (first four columns) along with previously characterized structures that overlap with each bin (fifth column)

RSCanner is designed to evaluate the base-pairing content of an existing RNA secondary structure, regardless of its origin. The input secondary structure can be derived from an *in silico* calculation (Tavareset al. 2021) or from an experimentally determined secondary structure, such as those derived from SHAPE experiments (Huston et al. 2021). During this process, RSCanner sequentially scans the input RNA secondary structure model, computes its relative BPC, and then assesses the conformational variability of the secondary structure across the RNA sequence. The program utilizes a secondary structure model that is expressed in connectivity table or dot-bracket format and a list of per-nucleotide SE values as inputs (Fig. 1A, Step 1). Both inputs can be directly obtained from databases like Rfam (Kalvari et al. 2021) or computed through third-party RNA secondary structure prediction programs with or without experimental constraints, e.g. Superfold (Smola et al. 2015) and RNAstructure (Reuter and Mathews 2010). Here, we have used Superfold to obtain the initial inputs for all RSCanner calculations. The BPC, defined here as the percent of double-stranded RNA, is calculated in sliding windows centered at each nucleotide across the entire RNA. SE values are smoothed by calculating the local median using the same sliding window approach (Fig. 1A, Step 2). SE is calculated from the probability of the formation of each base pair across all possible RNA conformations during structure prediction. High SE indicates that the RNA samples multiple conformations, while low SE suggests that it adopts one major conformation (Weeks 2021). Nucleotides with BPC values above (i.e. more structured than) and SE below (i.e. less conformationally dynamic than) a user-defined threshold are termed ‘structure counts’ (Fig. 1A, Step 3). By default, the program uses the global median (50th percentile) of both BPC and SE datasets as thresholds, but the user can adjust the stringency of these parameters by independently setting each cut-off value to a desired percentile. Finally, the frequency of structure counts (% structure content) is calculated in non-overlapping bins tiling the entire RNA, which is represented as a line plot with a 1D heatmap overlay (Fig. 1A, Step 4). In contrast, if a user wishes to identify conformationally variable regions with high BPC (e.g. interconverting secondary structures), one can visually examine the intermediate graphic outputs for regions where high BPC correlates with high SE (see Supplementary Fig. S1).

### 2.1 Graphic and text outputs

RSCanner produces several graphic outputs, illustrated here using a recently published Hepatitis C virus (HCV) genomic RNA structure (Wan et al. 2022). First, it outputs the calculated BPC and smoothed SE values for individual nucleotides in line plots (Supplementary Fig. S2A and B) and in accompanying text files. Second, it saves the nucleotides that pass both filtering thresholds (structure counts) into a text file and plots a histogram (Supplementary Fig. S2C). Last, RSCanner produces a line plot with 1D heatmap overlay (Fig. 1B), along with the corresponding data text file with binning windows in descending ordered of ‘% structure content’. Here, we note the top-scoring regions (highest % structure content) flagged in the HCV genome along with known RNA structural elements that overlap with them (Fig. 1C).

### 2.2 User-adjustable parameters

RSCanner allows the user to define several parameters. The sliding window size for BPC and/or SE smoothing and the bin size for the histogram can be changed to generate graphs of varying granularity. The lower and upper nucleotide bounds for visualization can also be altered, allowing the user to zoom in on specific regions of interest on the RNA. Furthermore, the stringency for filtering structure counts can be adjusted by individually varying the cut-off values for BPC and SE. For example, to increase the stringency, the user could increase the BPC cut-off value from the 25th to the 75th percentile (Supplementary Fig. S3A–C) and/or decrease the SE cut-off value from the 75th to 25th percentile (Supplementary Fig. S3A, D, and E).

## 3 Results

In addition to the HCV structure, we also applied RSCanner to other structured RNAs to illustrate its applicability to cases with and without experimental constraints: the HIV RNA genome, the SARS-CoV-2 Spike subgenomic RNA (sg-Spike), and the GRS1 long noncoding RNA (lncRNA GRS-1) (Supplementary Fig. S4). The SHAPE-informed HIV RNA secondary structure was obtained from its primary study (Siegfried et al. 2014), while the secondary structures for the other two RNAs were predicted with SuperFold (Smola et al. 2015) in the absence of experimental constraints.

## Supplementary Material

btad111_Supplementary_Data
