# Supplementary material for: RSCanner: rapid assessment and visualization of RNA structure content
Source: Bioinformatics. 2023 Mar 1;39(3):btad111. doi: 10.1093/bioinformatics/btad111 (PMC10017096; doi:10.1093/bioinformatics/btad111)
Supplement: btad111_Supplementary_Data [file btad111_supplementary_data.pdf]

Supplementary Data

Brief description of structural metrics used by RSCanner

Base pair content (BPC) is computed by calculating the percentage of double-stranded nucleotides within a given region (Tavares, et al., 2020).

Shannon Entropy: Shannon entropy for each nucleotide is calculated from base-pair probabilities derived from a secondary structure partition function calculation (Eq.1). Regions with low Shannon entropy are more likely to contain well-folded structures, with a single conformation (or few conformations) dominating the structure ensemble. High Shannon entropy regions are likely to sample multiple alternative conformations (Huynen, et al., 1997; Mathews, 2004; Weeks, 2021).

$$Eq.1 - Shannon\ Entropy_i = -\sum_{j=1}^{J} p_{i,j} \, log_{10}(p_{i,j})$$

$p_{i,j}$: *base pair probability for base pair ij*; J: *length of the RNA*

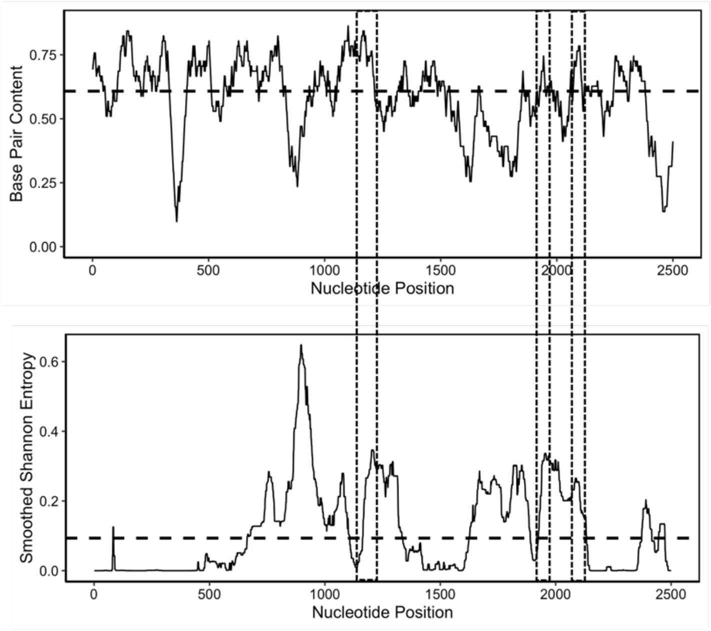

Supplementary Figure 1: RSCanner-guided identification of conformationally variable structured regions. Base Pair Content (BPC) (top panel) and Smoothed Shannon Entropy (bottom panel) were obtained with RSCanner for the first 2500 nt of the HCV genome. The cutoff values for BPC and SE (50th percentile) are shown as horizontal dashed lines. Regions displaying both high BPC and high SE are indicated in dashed line boxes and are likely to sample a variety of discrete secondary structures.

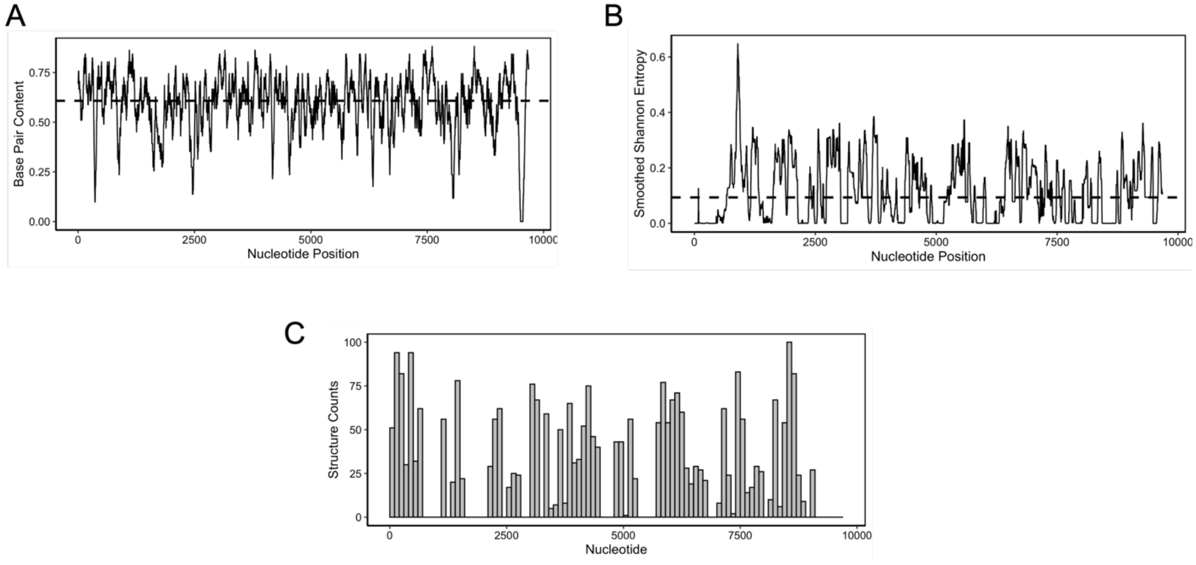

Supplementary Figure 2: Intermediate graphic outputs of RSCanner. The **(A)** Base Pair Content (BPC) and **(B)** Shannon Entropy (SE) are plotted against the center nucleotide (nt) of each sliding window. The cutoff values (default = 50th percentile) for BPC and SE are shown as dashed lines. **(C)** The frequency of nucleotides with BPC > cutoff and SE < cutoff (*structure counts*) is shown in 100-nt, non-overlapping bins.

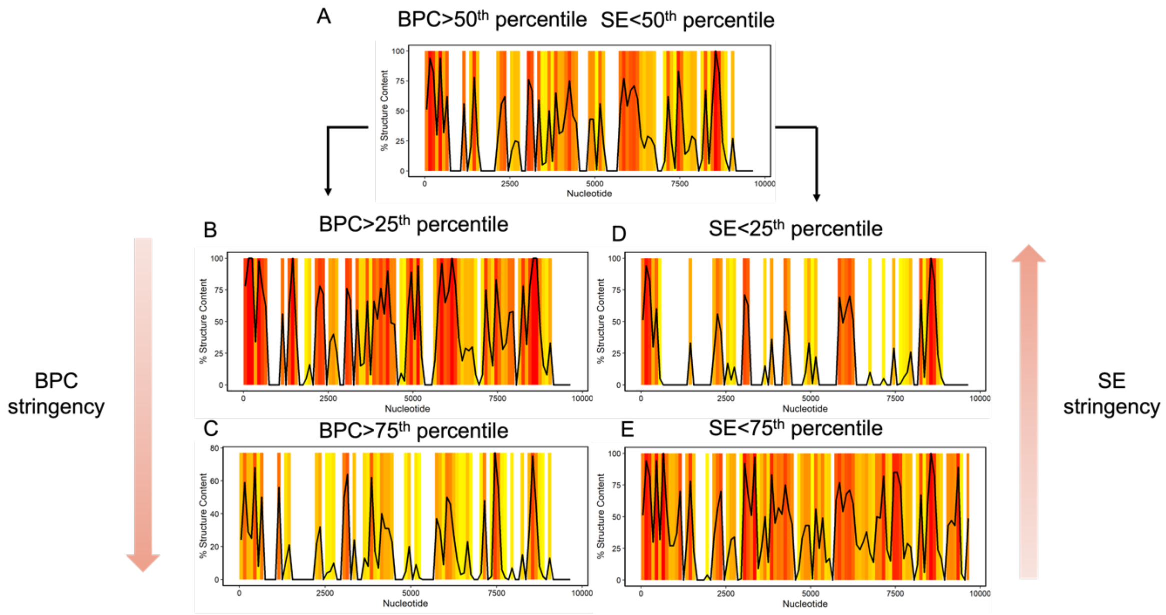

Supplementary Figure 3: Adjustment of RSCanner stringency parameters. (A) RSCanner heatmap plot on HCV Jc1 structure (Wan, et al., 2022) using the 50th percentile as the cutoff for both BPC and SE. **(B, C)** Different versions of the plot in **(A)** obtained by changing **(B)** the BPC cutoff value to the 25th percentile or **(C)** the 75th percentile and keeping the SE cutoff at the 50th percentile. **(D, E)** Different versions of the plot in **(A)** obtained by changing **(D)** the SE cutoff value to the 25th percentile or **(E)** the 75th percentile and keeping the BPC cutoff at the 50th percentile.

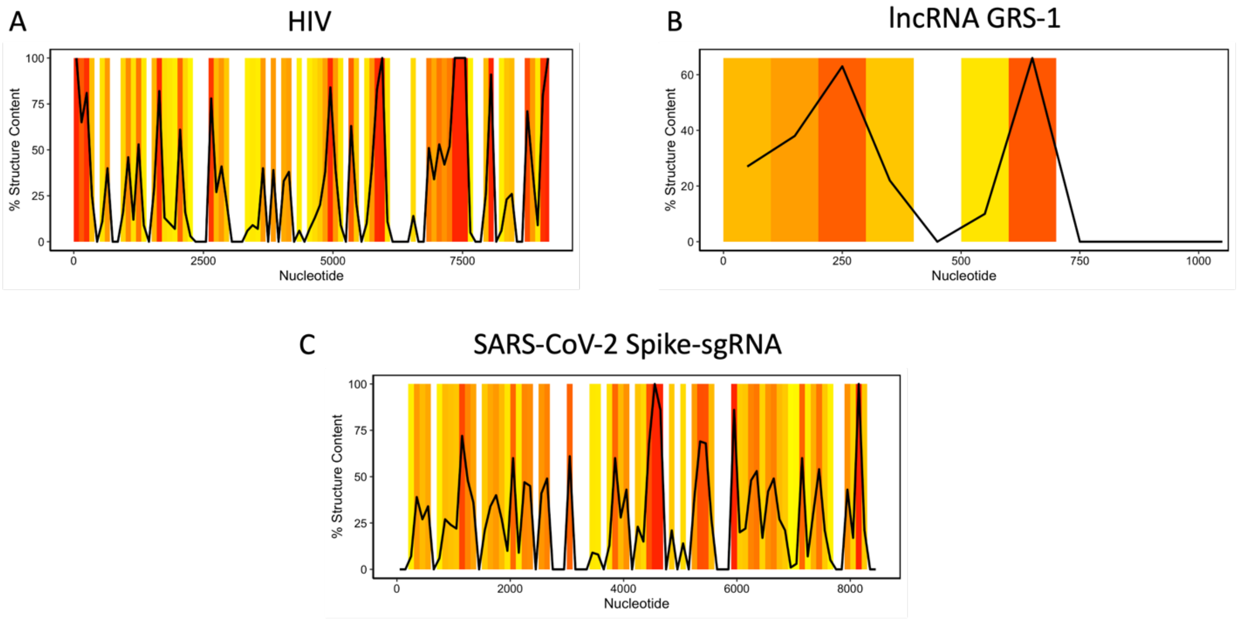

Supplementary Figure 4: Application of RSCanner to other RNAs. Final RSCanner heatmaps for the **(A)** HIV RNA genome (Siegfried, et al., 2014), **(B)** lncRNA GRS-1 (NCBI NR_109909), and **(C)** SARS-CoV-2 Spike-sgRNA (sequence derived from NCBI MN908947.3).

References:

Huynen, M., Gutell, R. and Konings, D. Assessing the reliability of RNA folding using statistical mechanics. *J Mol Biol* 1997;267(5):1104-1112.
Mathews, D.H. Using an RNA secondary structure partition function to determine confidence in base pairs predicted by free energy minimization. *RNA* 2004;10(8):1178-1190.
Siegfried, N.A.*, et al.* RNA motif discovery by SHAPE and mutational profiling (SHAPE-MaP). *Nat Methods* 2014;11(9):959-965.
Tavares, R.C.A.*, et al.* The global and local distribution of RNA structure throughout the SARS-CoV-2 genome. *J Virol* 2020.
Wan, H.*, et al.* The In Vivo and In Vitro Architecture of the Hepatitis C Virus RNA Genome Uncovers Functional RNA Secondary and Tertiary Structures. *J Virol* 2022;96(8):e0194621.
Weeks, K.M. SHAPE Directed Discovery of New Functions in Large RNAs. *Acc Chem Res* 2021;54(10):2502-2517.
